# Neutrophil‐secreted CHI3L1 exacerbates cardiac dysfunction and inflammation after myocardial infarction

**DOI:** 10.1096/fj.202401654R

**Published:** 2025-02-27

**Authors:** Jonah K. Stephan, Taylor Knerr, Zhen Gu, Hong Li, Kenneth R. Brittian, Sujith Dassanayaka, Richa Singhal, Yibing Nong, Steven P. Jones, Marcin Wysoczynski

**Affiliations:** ^1^ Center for Cardiometabolic Science, Christina Lee Brown Envirome Institute University of Louisville School of Medicine Louisville Kentucky USA

**Keywords:** heart failure, leukocytes, remodeling

## Abstract

Myocardial infarction (MI) triggers acute inflammation, marked by neutrophil infiltration. Although neutrophils are central to this response, the exact role of various neutrophil‐derived factors is not fully understood. Clinical studies have linked one such enigmatic factor, chitinase‐3 like‐1, to MI outcomes. Hence, we investigated its role in post‐MI remodeling. We found that chitinase‐3 like‐1 (CHI3L1) is upregulated after MI and secreted by activated neutrophils but does not directly affect neutrophil activity. To assess whether increased CHI3L1 influences ventricular remodeling, we subjected mice to non‐reperfused MI and administered recombinant CHI3L1. Increased CHI3L1 levels worsened ventricular remodeling. In contrast, CHI3L1‐deficient mice showed reduced ventricular remodeling after MI. To explore the underlying mechanisms, we assessed interactions with other cells known to be important in ventricular remodeling. Immunoprofiling of infarcted CHI3L1‐deficient mouse hearts revealed a faster decline in neutrophil and monocyte numbers, indicating quicker resolution of inflammation. These findings provide direct evidence that CHI3L1 exacerbates ventricular inflammation and remodeling following MI through gain‐ and loss‐of‐function approaches.

## Graphical abstract

CHI3L1 perpetuates non‐resolving inflammation and delays wound healing post‐MI. In response to myocardial injury, recruited neutrophils secrete Chitinase‐3 like‐1 (CHI3L1) to perpetuate non‐resolving inflammation and delay the wound healing response. As an effect, wound healing is delayed, as evidenced by scar expansion and exacerbated cardiac dysfunction. Created in BioRender. Stephan, J. (2025) https://BioRender.com/v83i423.

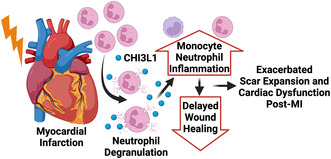

## 1 INTRODUCTION

A well‐orchestrated immune response after myocardial infarction (MI) clears necrotic tissue and promotes wound healing. Yet, chronic, overactive inflammation contributes to progressive changes in ventricular structure and function, resulting in heart failure (HF).^1^, ^2^, ^3^, ^4^, ^5^ Neutrophils are the first immune cells to infiltrate hearts after MI. They secrete granule‐stored proteolytic enzymes and cytokines, which facilitate extracellular matrix degradation and orchestrate subsequent monocyte and macrophage recruitment.^6^, ^7^, ^8^ Although neutrophils are necessary for infarct healing after MI, an overabundance of neutrophils is detrimental to ventricular remodeling.^9^, ^10^, ^11^ Yet, the specific roles of several neutrophil‐derived factors are not well understood.

Heart failure results in systemic plasma elevation of inflammatory biomarkers. Among the classical hallmarks of general inflammation like TNFα, other neutrophil‐specific inflammatory markers can be identified, including myeloperoxidase (MPO), neutrophil gelatinase‐associated lipocalin (NGAL), and neutrophil elastase (NE). More recently, the plasma of patients with cardiovascular disease was shown to contain a novel biomarker—chitinase‐3‐like‐1 (CHI3L1, in mice), also known as YKL‐40 or BRP‐39^12^, ^13^, ^14^, ^15^, ^16^, ^17^, ^18^, ^19^, ^20^, ^21^, ^22^ in humans. CHI3L1 is a glycoprotein and a member of mammalian chitinase‐like proteins that are associated with diseases characterized by inflammation, increased extracellular remodeling, and ongoing fibrosis; however, the exact function of CHI3L1 in heart failure is unknown.

Studies in patients with acute myocardial infarction show that CHI3L1 plasma levels increase compared to healthy age‐matched controls.^12^, ^13^, ^14^ Longitudinal studies in patients reveal that CHI3L1 levels acutely after MI negatively correlate with LV ejection fraction recovery.^14^, ^15^, ^16^, ^19^, ^21^, ^22^ Moreover, studies in patients with ischemic heart disease (IHD) show that the plasma level of CHI3L1 significantly associates with cardiovascular death and all‐cause mortality in the follow‐up period (2.6 years).^15^, ^19^ Although there is strong evidence that CHI3L1 is a reliable biomarker of inflammation, tissue remodeling, and cardiovascular disease, whether CHI3L1 contributes to the progression of heart failure remains untested.

In the current study, we established the role of CHI3L1 in the pathogenesis of MI‐induced heart failure. To address this knowledge gap, we used two approaches: chronic systemic administration of recombinant CHI3L1 protein and loss of function with a *Chil1* ^−/−^ (gene for CHI3L1) mouse. Indices of heart failure were assessed with echocardiography and histology. Given that CHI3L1 is associated with chronic inflammation, we assessed the magnitude and kinetics of immune cell infiltration in *Chil1* ^−/−^ mice after MI. Our study provides a primary assessment of the role of CHI3L1 in post‐MI ventricular remodeling and establishes its novel causal role.

## 2 MATERIALS AND METHODS

### 2.1 Mice

All animal experiments were performed following the Guide for the Care and Use of Laboratory Animals published by the U.S. National Institutes of Health and were approved by the University of Louisville Institutional Animal Care and Use Committee (Louisville, KY, USA). C57BL/6 mice at 14–22 weeks of age were purchased from Jackson Laboratories. *Chil1* ^−/−^ mice were a kind gift from Dr. Jack Elias. Upon arrival at the UofL facility, mice were housed in a pathogen‐free facility under a standard 12 h light/dark cycle with ad libitum access to food and water. Genomic DNA was isolated from tails using an EZ Tissue/Tail PCR Genotyping Kit (EZ BioResearch LLC, Saint Louis, MO, USA) according to the manufacturer's instructions.

### 2.2 Murine model of myocardial infarction

Adult (12–20 weeks) female mice were subjected to non‐reperfused, in vivo coronary artery ligation to induce heart failure, as described previously^23^, ^24^, ^25^, ^26^, ^27^, ^28^ and in accordance with the University of Louisville Institutional Animal Care and Use Committee. Mice were anesthetized using a combination of ketamine hydrochloride (50 mg/kg) and sodium pentobarbital (50 mg/kg), administered intraperitoneally. Mice were then intubated with PE‐60 tubing and mechanically ventilated, with 100% oxygen supplemented via the ventilator side port. Body temperature was maintained at 37.0 ± 0.5°C using a rectal thermometer interfaced with a servo‐controlled heat lamp. Using a sterile technique, mice were subjected to a thoracotomy, and the left coronary artery was visualized with the aid of a dissecting microscope and permanently occluded with 7–0 silk suture. After ligation, the chest and skin were closed using 4–0 silk and polyester monofilament sutures, respectively. Mice were given 5 mg/kg ketoprofen, subcutaneously, for analgesia. Upon recovery of spontaneous respiration, the intubation tube was removed, and mice were allowed to recover in a temperature‐controlled area supplemented with 100% oxygen. Mice were given additional doses of ketoprofen (5 mg/kg) by 24 and 48 h post‐operatively. For all studies involving myocardial infarction, mouse exclusions were based on a lack of transmural infarct in mid‐papillary heart sections stained with Masson's trichrome.

### 2.3 Echocardiography

Transthoracic echocardiography of the left ventricle was performed as previously described^23^, ^24^, ^25^, ^26^, ^27^, ^28^, ^29^, ^30^, ^31^, ^32^, ^33^, ^34^, ^35^, ^36^ with a Vevo 3100 echocardiography system. Body temperature was maintained at 36.5–37.5°C using a rectal thermometer interfaced with a servo‐controlled heat lamp. Mice were anesthetized with 2% isoflurane and then maintained under anesthesia with ~1.5% isoflurane. Using the Vevo rail system, the mouse was placed supine on an examination board interfaced with the Vevo 3100. Next, depilatory cream was applied to the mouse's chest and wiped clean to remove fur from the chest. The MX550D (25–55 MHz) scan head was used to obtain 2D images (250 fps) of the parasternal long axis; M‐mode images were also acquired from this position. The probe was then rotated 90° to acquire short axis views. Beginning at the base and moving apically, serial 2D images were taken every millimeter. All measurements were taken with the aid of the Vevo 3100's rail system to maintain probe placement and allow for precise, minute adjustments of probe position along the long axis. Left ventricular diameters during diastole and systole (LVIDd and LVIDs) were determined from long‐axis M‐modes along with heart rate (HR). Left ventricular fractional shortening (%FS) was calculated as: ((LVIDd‐LVIDs)/LVIDd) × 100%. Diastolic and systolic volumes were determined by applying Simpson's rule of discs to the serially acquired short‐axis images. Stroke volume (SV) was calculated as: diastolic volume—systolic volume. Ejection fraction was calculated as: (SV/Diastolic Volume) × 100%. Cardiac output was determined by: SV × HR.

### 2.4 Histology

After the final echocardiography, hearts were arrested in diastole with saturated KCl and CdCl_2_ (100 mmol/L). The heart was excised, the aorta was then cannulated, and the heart was perfused with PBS followed by 10% buffered formalin at 75 mm Hg while the LV was perfused through an apical cannula with formalin at 8 mm Hg (to preserve overall spherical geometry). Hearts were then cut into 2 mm cross‐sectional slices and processed for paraffin embedding. Slices were cut into 4 μm sections for histology and immunofluorescent staining. LV area, risk area, LV cavity area, and infarct area were measured in Masson's trichrome stained sections as previously described.^37^, ^38^ LV dilation and wall thinning were measured by determining the expansion index as previously described.^37^, ^38^ The expansion index was calculated as (LV cavity area/total area) × (non‐infarcted region wall thickness/risk region wall thickness). The risk area was defined as the transmural area between the furthest outer lateral progressions of the scar, which was defined by Masson's trichrome staining. Viable myocardium in the risk area was determined as the difference between risk and scar areas. Images were acquired digitally, and areas were measured using ImageJ (1.54v).

### 2.5 Flow cytometric analysis of tissues

At 2, 3, and 5 days post‐MI, tissues were collected for quantification of cell types by flow cytometric analysis. At 2 days post‐MI, peripheral blood, spleen, bone marrow, and hearts were collected for flow. Peripheral blood was collected via retro‐orbital bleeding and kept on ice in 200 μL Microvette® collection tubes to prevent coagulation. Peripheral blood was stained by adding 100 μL of blood to round bottom tubes, then adding 100 μL of 2× concentration antibodies. Cells were stained for 30 min on ice, then erythrocytes were lysed by adding 2 mL of 1× Lysis Buffer (BD Bioscience, USA) and incubating at room temperature for 10 min. Following lysis, 2 mL of 1× PBS (Gibco, Grand Island, NY, USA) was added to each tube to inactivate lysis buffer, then cells were pelleted at 600*g* for 10 min at 4°C. Peripheral blood samples were subjected to another wash cycle, then resuspended in 1 mL of 1× PBS then analyzed by flow cytometry. Leukocyte per μL of blood values were acquired by Hemavet®. Bone marrow was acquired by flushing full‐length femurs with 20 mL of ice‐cold 1× PBS (Gibco, Grand Island, NY, USA). Cells were then pelleted at 600*g* for 10 min at 4°C and resuspended in 1–2 mL of ice‐cold 1× PBS. Preparation of cells for analysis by flow cytometry followed a similar procedure to peripheral blood, except no lysis of erythrocytes was necessary after staining on ice. Bone marrow cells were counted in Türk's Solution manually with a hemacytometer to acquire cells/femur. Splenocytes were isolated by chopping with a razor blade and smashing spleens with the plunger of a 1 mL syringe. Cells were strained with a 70 μm cell strainer (Corning, USA), then pelleted and resuspended in 5–10 mL of 1× PBS (Gibco, Grand Island, NY, USA). The preparation of cells for flow cytometry is identical to bone marrow. Cells were counted manually in Türk's solution and normalized to mg of tissue. Hearts were digested to provide a single‐cell suspension for analysis by flow cytometry. Hearts were collected and perfused with 1× PBS via Langendorff Perfusion for 5–10 min to remove all peripheral blood. To generate a single‐cell suspension, hearts were thoroughly minced with a razor blade and digested with 12,000 U of Type I Collagenase (Worthington Biochemical, Lakewood, NJ, USA) as previously described.^39^ Residual collagenase was washed out by adding 1× PBS (Gibco, Grand Island, NY, USA) and pelleting cells at 600*g* for 10 min at 4°C. Immune cells were separated from debris and cardiomyocytes using a 30% Percoll gradient and centrifuged at 600 × *g* for 30 min at 4°C. The supernatant containing debris and cardiomyocytes was removed via suction, and pelleted immune cells were washed with 1× PBS (Gibco, Grand Island, NY, USA) twice to remove any residual Percoll. After washing, cells were stained following a procedure identical to bone marrow and spleens. Cells were counted in the absence of Türk's Solution and normalized to mg of tissue.

### 2.6 Isolation and culture of macrophages

C57BL/6 mice were euthanized by CO_2_ exposure. Tibia and femur bones were flushed with ice‐cold 1× PBS using a 21‐gauge needle. The collected bone marrow cell pellet was suspended in a growth medium consisting of DMEM/F12 (Invitrogen, Waltham, MA, USA), 10% FBS (Seradigm, VWR), 40 ng/mL M‐CSF (Peprotech, Cranbury, NJ, USA), and 100 U/mL penicillin–streptomycin (Thermo Fisher Scientific). A single‐cell suspension was plated in the tissue culture flask. After 24 h, floating cells were removed, and adherent cells were expanded for 7–10 days with media change every other day. Macrophage purity was determined by co‐expression of CD11b and F4/80 markers, evaluated with flow cytometry. To induce a pro‐inflammatory (M1) phenotype, macrophages were cultured for 24 h in the presence of 100 ng/mL LPS (Peprotech, Cranbury, NJ, USA) and 50 ng/mL INF‐γ (Peprotech, Cranbury, NJ, USA); to induce an anti‐inflammatory (M2) phenotype, macrophages were cultured for 24 h in the presence of 10 ng/mL IL‐4 (Peprotech, Cranbury, NJ, USA) and 10 ng/mL IL‐13 (Peprotech, Cranbury, NJ, USA). Peritoneal macrophages were isolated from peritoneal lavages of C57BL/6 mice. Peritoneal cavities were lavage with 3 mL of 1× DPBS−/−, and cells were allowed to adhere to the bottom of a 6‐well plate in DMEM F/12 + 10% FBS. After 1 h, non‐adherent cells were washed away leaving adherent macrophages as previously described.^40^ Cells were then cultured in DMEM F/12 + 10% for 24 h in the presence of M1 or M2 polarizing cytokines. Polarization of macrophages was done concurrently with recombinant CHI3L1 (R&D Systems, Minneapolis, MN, USA) stimulation at 500 ng/mL.

### 2.7 Bone marrow neutrophil isolation

Bone marrow cells were isolated from tibias and femurs as described above. Bone marrow neutrophils were isolated with a neutrophil isolation kit (Miltenyi Biotec), according to the manufacturer's recommendations. Briefly, neutrophils were isolated by depletion of non‐target cells. Non‐target cells were labeled with a cocktail of biotin‐conjugated monoclonal antibodies, as the primary labeling reagent, followed by anti‐biotin monoclonal antibodies conjugated to MicroBeads as a secondary labeling reagent. The magnetically labeled non‐target cells were depleted by retaining them on the LS MACS Column in the magnetic field of a MACS separator, while unlabeled neutrophils ran through the column. The purity of the neutrophil sort was evaluated by CD11b and Ly6G co‐expression with flow cytometry, as described.^39^, ^41^

### 2.8 Cardiac fibroblast isolation

Mice were euthanized by sodium pentobarbital injection (100 mg/kg i.p.). The hearts were excised, washed in room temperature 1× PBS, minced into small pieces, and enzymatically dissociated with Collagenase II (5 mg/mL in PBS; Worthington) with gentle agitation for 45 min at 37°C. After Collagenase II inactivation with DMEM/F12 medium containing 10% FBS, cells were centrifuged at 600 × *g* for 10 min. The collected cell pellet was suspended in a growth medium consisting of DMEM/F12 (Invitrogen, Waltham, MA, USA), 10% FBS (Seradigm, VWR), 20 ng/mL bFGF (Peprotech, Cranbury, NJ, USA), ITS (insulin/transferrin/selenium), glutamine, and 100 U/mL penicillin–streptomycin (Thermo Fisher Scientific). Cells were seeded in T75 cell culture flasks for 2 h. Subsequently, flasks were washed in PBS, and adherent cells were cultured in growth media upon 70% confluence with media change every other day. Some fibroblasts were exposed to type 1 and type 2 cytokines to mimic the inflammatory and pro‐reparative cardiac environment after MI. Fibroblasts cultured with 100 ng/mL LPS (Peprotech, Cranbury, NJ, USA) and 50 ng/mL INF‐γ (Peprotech, Cranbury, NJ, USA) were labeled as Fibs 1, and 10 ng/mL IL‐4 (Peprotech, Cranbury, NJ, USA) and 10 mg/mL IL‐13 (Peprotech, Cranbury, NJ, USA) as Fibs 2, respectively. Control fibroblasts were labeled as Fibs 0.

### 2.9 Bone marrow B cell isolation

Bone marrow cells were isolated from tibias and femurs and subjected to B cell isolation with a B cell isolation kit (Miltenyi Biotec), according to the manufacturer's recommendations.

### 2.10 Spleen T cell isolation

Splenocytes were isolated by mincing with a razor blade and smashing spleens with a 1 mL syringe piston. Cells were strained with a 70 μm cell strainer (Corning, USA) to remove large tissue pieces, then pelleted and suspended in 1× PBS (Gibco, Grand Island, NY, USA). Single‐cell suspensions were subjected to T cell isolation with a T cell isolation kit (Miltenyi Biotech), according to the manufacturer's recommendations.

### 2.11 CHI3L1 quantification in heart tissue and isolated cells

At 2, 7, and 35 days after sham operation or MI, mice were euthanized. The hearts were excised and perfused with cold PBS for 10 min to wash out peripheral blood. Subsequently, left ventricles were dissected into infarcted and remote regions for CHI3L1 quantification. For the isolation of total protein extracts from cardiac tissue or isolated cells, flash‐frozen tissue specimens or cell pellets were suspended in RIPA lysis buffer (Thermo Fisher Scientific) containing 1 × Halt™ Protease and Phosphatase Inhibitor Cocktail (Thermo Fisher Scientific) at a ratio of 40 μL per mg of tissue. Tissue segments were then mechanically dissociated on ice using an electric tissue homogenizer. Samples were then incubated on ice for 30 min with periodic agitation and finally centrifuged at 14 000 × *g* for 15 min at 4°C to remove insoluble material. Total protein lysates were quantified for total protein content using a Pierce™ BCA Protein Assay Kit (Thermo Fisher Scientific) according to the manufacturer's instructions. Subsequently, the protein lysates were analyzed for CHI3L1 content with ELISA according to the manufacturer's recommendations (R&D Systems, Minneapolis, MN, USA).

### 2.12 Neutrophil degranulation

Neutrophils were sorted via negative selection from the bone marrow of *Chil1* ^*+/+*^ and *Chil1* ^*−/−*^ mice. After sorting, neutrophils were resuspended to 1 × 10^6^ cells/mL in DMEM F/12 + 0.5% BSA and plated in 12‐well plates (Corning, USA) and allowed to equilibrate at 37°C (5% CO_2_) for 15 min. Following equilibration, cells were stimulated with ranging doses of PMA (Sigma‐Aldrich, St. Louis, MO, USA) or fMLF (Cayman Chemical, Anna Arbor, MI, USA) for 1 h at 37°C (5% CO_2_). After 1 h of stimulation, conditioned media were collected from each well, and centrifuged at 600 × *g* for 10 min at 4°C to pellet residual cells, and supernatants were transferred to new tubes and stored long‐term at −80°C. Quantification of myeloperoxidase (MPO), neutrophil gelatinase‐associated lipocalin‐2 (NGAL), and MMP‐9 was performed via ELISA (R&D Systems, Minneapolis, MN, USA), following company‐recommended procedure.

### 2.13 Co‐culture of macrophages with neutrophils

Bone marrow neutrophils from *Chil1* ^−/−^ and their control littermates were stimulated with 10 μM A32187 ionophore (Cayman Chemical, Anna Arbor, MI, USA) for 20 min. After stimulation, residual ionophore was washed out with 1× PBS (Gibco, Grand Island, NY, USA) by pelleting cells at 600 × *g* for 10 min at 4°C. Pelleted cells were resuspended in DMEM F/12 + 10% FBS supplemented with 100 U/mL penicillin–streptomycin and added directly to co‐culture with macrophages at a ratio of 10:1 neutrophil to macrophages. After 24 h in culture, the expression of macrophage inflammatory markers was quantified by RT‐qPCR.

### 2.14 RNA isolation and real‐time qPCR

Total RNA was isolated from bone marrow‐derived macrophages with a RNeasy Mini Kit (Qiagen) following the manufacturer's recommendations. Conversion of isolated mRNA to cDNA was conducted with the High‐Capacity cDNA Reverse Transcription Kit (Applied Biosystems, USA) and subsequently diluted to 2.5–4.0 ng/μL. The expression of pro‐inflammatory (*Il1a, Il1b, Tnfa, Il12b*), pro‐reparative (*Chil3, Chil4, Arg1, Fizz1*), and housekeeping genes (*B2m* and *Hprt*) was quantified with qPCR (Table 1). RT‐qPCR was carried out on a Quantstudio 5 (Applied Biosystems) in a 10 μL reaction containing 2 μL of cDNA, 5 μL SYBR Green Master Mix (Applied Biosystems), and relative mRNA expression was evaluated via the comparative Ct method as described.^39^ Several measures were taken to avoid contaminating genomic DNA amplification, including (1) employing a gDNA Eliminator column included with the RNeasy Mini Kit (Qiagen) during RNA isolation, (2) using primers designed to cross exon‐exon junctions, and (3) utilizing no‐template controls with each primer set.

**TABLE 1:** RT‐qPCR primer sequences.

Target	Forward primer sequence	Reverse primer sequence
*Hprt*	AGGACCTCTCGAAGTGTTGG	AGGGCATATCCAACAACAAAC
*B2m*	GATCACATGTCTCGATCCCAGTAG	CATACGCCTGCAGAGTTAAGC
*Il1a*	AGGGAGTCAACTCATTGGCG	TGGCAGAACTGTAGTCTTCGT
*Il1b*	TCGTGCTGTCGGACCCATAT	GGTTCTCCTTGTACAAAGCTCATG
*Tnfa*	GGGCCACCACGCTCTTC	CCTCCACTTGGTGGTTTGCT
*Il12b*	TTCAACATCAAGAGCAGTAGCAG	CCTGGCAGGACACTGAATACTT
*Chil3*	CCAGCAGAAGCTCTCCAGAAG	TGGTAGGAAGATCCCAGCTGTA
*Chil4*	AATCCACTTTGAACCACATTCCA	CCTGAATATAGTCGAGAGACTGAG
*Arg1*	TTTTAGGGTTACGGCCGGTG	CCTCGAGGCTGTCCTTTTGA
*Fizz1*	CCTGCTGGGATGACTGCTACT	GCAGTGGTCCAGTCAACGA

### 2.15 Single‐cell RNA sequencing and bioinformatics analysis

Three female C57BL/6J (The Jackson Laboratory) mice were injected with G‐CSF (Filgrastim 100 μg/kg/day) or Vehicle (1× PBS) for three days. Twenty‐four hours after the final injection, bone marrow cells from each mouse were collected and combined within the G‐CSF and Vehicle groups, respectively. Cells were flushed from femurs with cold PBS + 0.04% BSA, pelleted at 600 × *g* for 10 min (4°C), and resuspended in PBS + 0.04% SA to obtain 1 × 106^6^ cells/mL. Cell counts were conducted in Tuerk's solution to exclude erythrocytes, and viability was assessed using Trypan Blue. Cells were processed and captured using the 10X Chromium iX Single Cell Instrument, and libraries were prepared using Chromium Next GEM Single Cell 3′ Reagent Kits v3.1 (Dual Index). Sequencing was performed on the Illumina NextSeq 2000 platform using a P3 100‐cycle reagent kit with a P3 flow cell. The samples were initially processed using the 10X Genomics CellRanger suite version 3.1.0.^42^ The raw base call files were demultiplexed into FASTQ files using the cellranger mkfastq command, and sequence quality was determined using FastQC. The Cell Ranger count function was used to align the raw reads to the mm10 genome and generate gene counts for each cell. The count matrix was imported as a Seurat object and analyzed using Seurat v4.^43^, ^44^, ^45^ Data was filtered based on the following settings—Mitochondrial percentage = 30, Minimum number of features per cell ≥200, and Maximum number of features per cell ≤5000. Data were normalized using the LogNormalize function, and a scale factor of 10 000 and 2000 highly variable genes were identified using the methodology of Seurat developers.^43^, ^44^, ^45^ Seurat FindNeighbors and FindClusters functions were utilized to generate an integrated UMAP with dimensions set to 10 and resolution set to 0.3. Cell Clusters were identified based on distinct gene expression profiles obtained from the Seurat FindMarkers function.^43^, ^44^, ^45^

### 2.16 Statistical analysis

Results are shown as means or means ± SEM. Statistical analysis was conducted using a one‐tailed Student's *t*‐test to compare two groups, one‐way or two‐way ANOVA for three or more groups. All statistical analyses were performed using GraphPad Prism, version 10 (GraphPad Software, Boston, MA, USA). Differences were considered statistically significant if *p* < .05.

## 3 RESULTS

### 3.1 CHI3L1 is increased in hearts after MI

**FIGURE 1:**
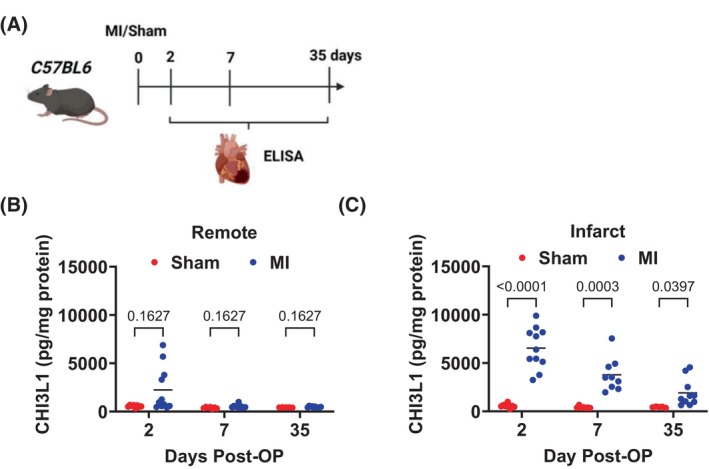
CHI3L1 is increased in infarcted hearts after MI. Mouse hearts were collected at 2, 7, and 35 days post‐MI and sham operation (A). Remote and infarct regions of LV were assessed for CHI3L1 expression with ELISA (B and C). Multiple *t*‐tests.

Given that CHI3L1 is elevated in patients with MI, and the plasma level correlates with adverse clinical outcomes of heart failure,^12^, ^13^, ^14^, ^15^, ^16^, ^17^, ^18^, ^19^, ^20^, ^21^ we determined if and in what region CHI3L1 may be upregulated post‐MI. To test this, we harvested infarcted and remote regions of LV from mice at 2, 7, and 35 days post‐MI, and quantified CHI3L1 by ELISA (Figure 1A). We found that the CHI3L1 protein expression in hearts is unchanged in Remote LV areas at 2, 7, and 35 days after MI (Figure 1B)—though some mice (4 out of 11) showed elevated expression of CHI3L1 in remote regions of LV (Figure 1B). This may be due to morphologically poorly defined infarcted regions in hearts at early time points after MI and our limitations in accurately dissecting the corresponding regions, rather than elevated CHI3L1 in distal regions of the infarct. Conversely, CHI3L1 levels increased in infarcted regions in response to MI. The highest CHI3L1 expression transpired in hearts at 2 days after MI and gradually declined at 7 and 35 days but remained elevated in infarcted regions of LV compared to sham hearts (Figure 1C). Together, these data suggest that CHI3L1 protein expression is elevated in infarcted regions of the hearts after MI in the acute and chronic phases, but the expression of CHI3L1 in non‐infarcted tissue remained unchanged.

### 3.2 Administration of exogenous CHI3L1 exacerbates cardiac dysfunction and remodeling after MI

**FIGURE 2:**
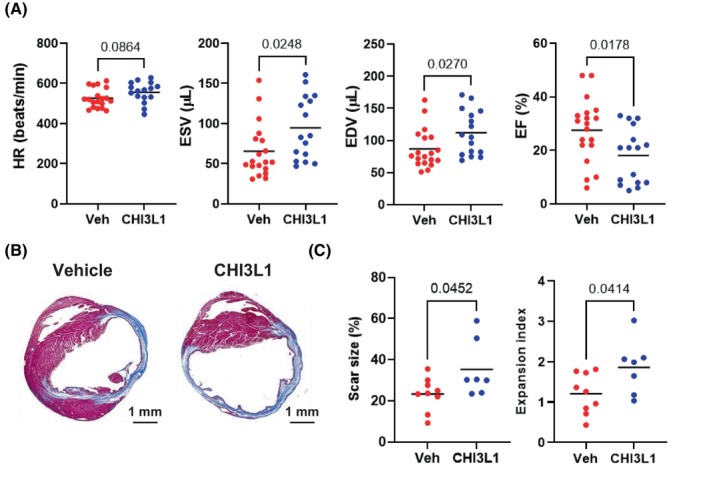
Systemic administration of exogenous CHI3L1 exacerbates LV dysfunction and remodeling after MI. C57BL/6 mice were subjected to MI and injected with vehicle (Veh) or exogenous CHI3L1 (250 μg/kg/day) at the time of surgery and then every other day. At 35 days post‐MI, heart rate (HR), end‐systolic volume (ESV), end‐diastolic volume (EDV), and ejection fraction (EF) were measured via echocardiography (A). Student's *T*‐test. At 35 days post‐MI, heart sections were stained with trichrome (B). Scar size and expansion index were quantified with morphometric analysis (C). Student's *t*‐test.

To test the role of excess CHI3L1 in ventricular remodeling after MI, C57BL/6 mice were injected with either vehicle or exogenous CHI3L1 at the time of MI surgery and then every other day for 35 days. At 35 days, cardiac function was measured with echocardiography. We found that mice injected with exogenous CHI3L1 exhibited increased end‐systolic (ESV) and end‐diastolic (EDV) volumes after MI (Figure 2A), indicating an increase in left ventricular dilation. Furthermore, ejection fraction was further decreased in mice injected with exogenous CHI3L1 compared to vehicle‐injected mice (Figure 2A). To gain more insights into ventricular structure after CHI3L1 administration, mouse heart sections were stained with Masson's Trichrome (Figure 2B). We found that CHI3L1 administration increased scar size and expansion index (Figure 2C). These data suggest that further increasing CHI3L1 after MI contributes to adverse ventricular remodeling and pump dysfunction.

### 3.3 Neutrophils produce CHI3L1

Prior reports show that CHI3L1 is produced by various cells including neutrophils, macrophages, fibroblasts, and vascular cells; however, the direct contribution of each cell type to elevated cardiac CHI3L1 after MI has not been tested. Our bone marrow scRNA seq analysis revealed that *Chil1* gene expression colocalizes with the neutrophil marker *Ly6g*, but not with other relevant cell types including monocytes/macrophages, B cells, and T cells (Figure S1A). Next, we quantified the protein expression of CHI3L1 in common cell types in the heart, such as neutrophils, macrophages, fibroblasts, endothelial cells, and cardiomyocytes. We isolated each of these cell types and induced various relevant phenotypes, such as M0, M1, and M2 macrophages and fibroblasts. Neutrophils expressed more CHI3L1 per mg of protein by ELISA than each of the other cell types (Figure S1B). Upon activation, neutrophils secrete prepackaged proteins. We found that neutrophils stimulated with PMA, A23187 ionophore, but not with LPS, rapidly secrete CHI3L1 into the media (Figure S1C). Because neutrophils amass in large numbers in the heart acutely post‐MI and express significantly more CHI3L1 than other types of cells, we expect that they are the predominant contributors to CHI3L1 in the heart post‐MI. Collectively, these data show that CHI3L1 increased in the ischemic zone of the heart acutely post‐MI and is derived from neutrophils. Next, we determined the contribution of CHI3L1 to autocrine signaling in neutrophils.

### 3.4 CHI3L1 has no effect on neutrophil activation and recruitment after MI

**FIGURE 3:**
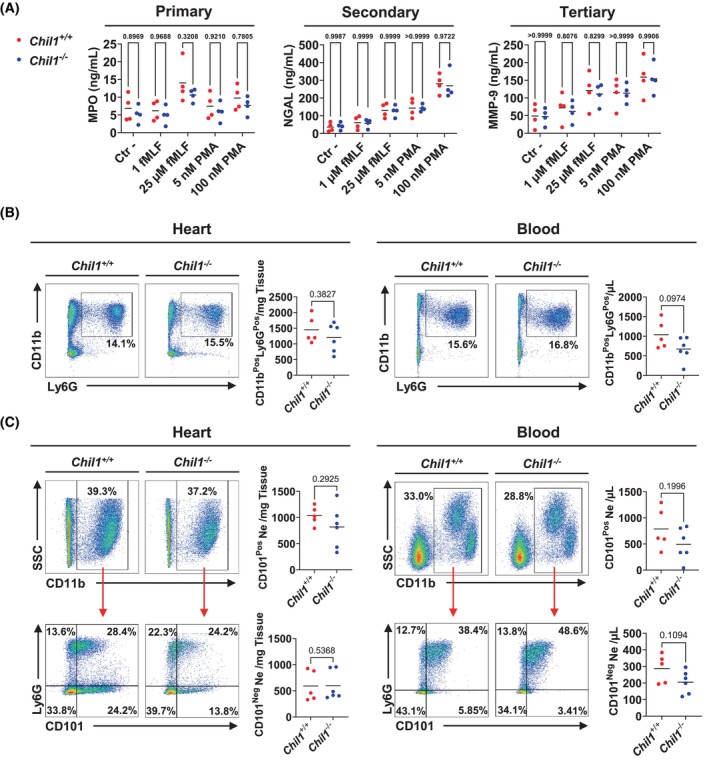
CHI3L1 has no effect on neutrophil activation, recruitment, and mobilization after MI. Chil1^+/+^ and Chil1^−/−^ neutrophils were stimulated with fMLF or PMA, and granule components were quantified in conditioned media by ELISA (A). Two‐way ANOVA. Chil1^+/+^ and Chil1^−/−^ mice were subjected to MI. At 2 days post‐MI, CD11b^Pos^Ly6G^Pos^ neutrophils were quantified in hearts and blood (B) and further stratified into CD101^Neg^ immature and CD101^Pos^ mature neutrophils (C). Student's *t*‐test.

Neutrophils secrete copious amounts of cytokines, chemokines, and bioactive lipids that recruit and activate new waves of neutrophils to the site of inflammation. To determine the contribution of CHI3L1 to feed‐forward neutrophil recruitment and activation, we sorted bone marrow neutrophils from *Chil1* ^*+/+*^ and *Chil1* ^*−/−*^ mice and stimulated them with PMA and fMLF to induce degranulation. Degranulation was assessed by quantifying granule proteins in conditioned media via ELISA. Conditioned media from *Chil1* ^*+/+*^ and *Chil1* ^*−/−*^ neutrophils showed no difference in the primary granule components (Figure 3A) myeloperoxidase (MPO), the secondary granule protein neutrophil‐gelatinase associated lipocalin‐2 (NGAL), or the tertiary granule protein matrix metalloproteinase‐9 (MMP‐9), suggesting that CHI3L1 is unlikely to play an autoregulatory role in neutrophil activation to secrete granule components. To further test this notion, we subjected *Chil1* ^*+/+*^ and *Chil1* ^*−/−*^ mice to MI and quantified neutrophils in the heart, bone marrow, peripheral blood, and spleen at 2 days post‐MI. We found CHI3L1 has no effect on the recruitment of neutrophils to the heart or mobilization in the blood at 2 days post‐MI (Figure 3B). Furthermore, we saw no differences in the accumulation of neutrophils in the bone marrow or spleen (Figure S2A,B). These data further support the observation that CHI3L1 has no effect on autocrine/paracrine neutrophil activation, recruitment, or production. Because the mass recruitment of neutrophils to the heart post‐MI involves the mobilization and recruitment of immature neutrophils, canonically identified as CD101^Neg^, we determined if CHI3L1 contributed to the differential recruitment of subsets of neutrophils. We immunoprofiled neutrophils in the heart, peripheral blood, bone marrow, and spleen cells for the expression of the maturity marker CD101. Again, we did not observe any difference in the proportion of CD101^Pos^ and CD101^Neg^ neutrophils in the heart or peripheral blood between *Chil1* ^*+/+*^ and *Chil1* ^*−/−*^ (Figure 3C), neither in the bone marrow nor spleen (Figure S2C,D). These data suggest that CHI3L1 does not affect neutrophil dynamics at 2 days after MI. Thus, in the next experiments, we explored the role of CHI3L1 on the dynamics of subsequent inflammatory phases after MI.

### 3.5 CHI3L1 perpetuates inflammation in infarcted hearts

**FIGURE 4:**
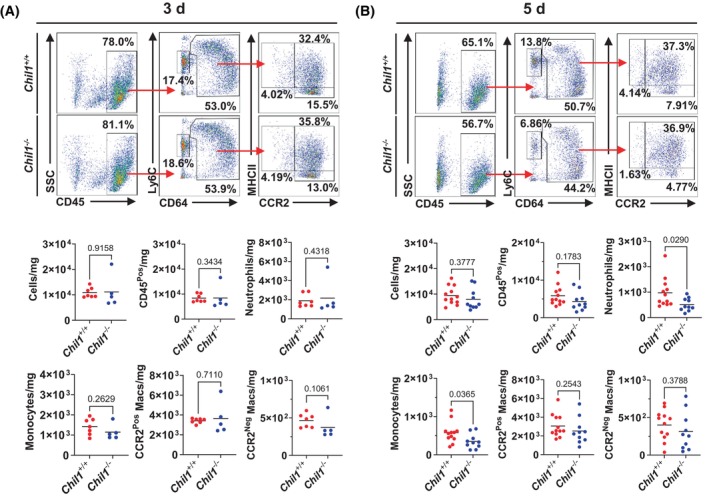
CHI3L1 perpetuates ventricular inflammation after MI. Chil1^+/+^ and Chil1^−/−^ mice were subjected to MI. At 3 and 5 days post‐MI, hearts were perfused, digested, and probed with fluorescently labeled antibodies to detect neutrophils (CD45^Pos^CD64^Neg^Ly6C^Low^), monocytes (CD45^Pos^CD64^Pos^MHCII^Neg^CCR2^Pos^), and macrophages (CD45^Pos^CD64^Pos^MHCII^Pos^CCR2^Pos^ and CD45^Pos^CD64^Pos^MHCII^Pos^CCR2^Neg^). Each cell type was normalized to cells/mg isolated from each heart at 3 days (A) and 5 days post‐MI (B). Student‘s *t*‐test.

Neutrophils are fast responders and appear at the site of inflammation within minutes to hours following MI. Numerous neutrophil‐secreted factors orchestrate the subsequent inflammatory response, including monocyte recruitment and their descendant macrophage differentiation and polarization. Thus, we elucidated the impact of neutrophil‐secreted CHI3L1 on monocyte and macrophage dynamics. To test this, we subjected *Chil1* ^*+/+*^ and *Chil1* ^*−/−*^ mice to MI and quantified monocytes and macrophages in the heart at 3 and 5 days post‐MI. We found that at 3 days post‐MI, there were no differences in monocytes, neutrophils, CCR2^Pos^ monocyte‐derived macrophages, or CCR2^Neg^ monocyte‐derived macrophages (Figure 4A), further indicating that CHI3L1 does not contribute to the initiation of inflammation post‐MI. At 5 days post‐MI, we found that recruited neutrophils were decreased in our *Chil1* mice^*−/−*^ relative to *Chil1* ^*+/+*^ controls (Figure 4B), and there were trends in decreasing overall leukocytes (CD45^Pos^) and monocytes in *Chil1* ^*−/−*^ mice (Figure 4B). Collectively, CHI3L1 does not contribute to the initiation of inflammation, as indicated by no differences in neutrophil numbers at 2 days post‐MI (Figure 3B) and overall leukocytes at 3 days (Figure 4B); however, the differences observed in neutrophil and monocyte accumulation at 5 days indicate that CHI3L1 antagonizes the resolution of inflammation (Figure 4B).

### 3.6 CHI3L1 does not impact macrophage polarization

To determine if CHI3L1 influences macrophage polarization, we stimulated pro‐inflammatory M1 and pro‐reparative M2 polarization of bone marrow‐derived macrophages (BMDMs) and peritoneal macrophages in the presence or absence of recombinant CHI3L1. We found that BMDMs exhibited increased expression of pro‐inflammatory cytokines in response to treatment with IFN‐γ and LPS, consistent with an M1 phenotype (Figure S3A); however, CHI3L1 did not affect expression of pro‐inflammatory cytokines except for *Il12b* (Figure S3A). Furthermore, we showed that the presence of CHI3L1 had no effect on the IFN‐γ/LPS‐induced pro‐inflammatory gene expression in peritoneal macrophages (Figure S3B). BMDMs exhibited upregulation of pro‐reparative genes in response to treatment with IL‐4/Il‐13, as consistent with M2 polarization (Figure S3C), but we did not observe an impact on gene expression by CHI3L1 (Figure S3C). Similarly, CHI3L1 had no effect on M2 gene expression in peritoneal macrophages (Figure S3D). These data suggest that CHI3L1 promotes non‐resolving inflammation independent of direct signaling to macrophages. Because CHI3L1 promotes non‐resolving inflammation in vivo, and prolonged inflammation antagonizes scar formation post‐MI,^2^, ^4^, ^5^ we next tested the role of CHI3L1 in ventricular remodeling and function after MI.

### 3.7 CHI3L1 exacerbates cardiac remodeling and dysfunction after MI

**FIGURE 5:**
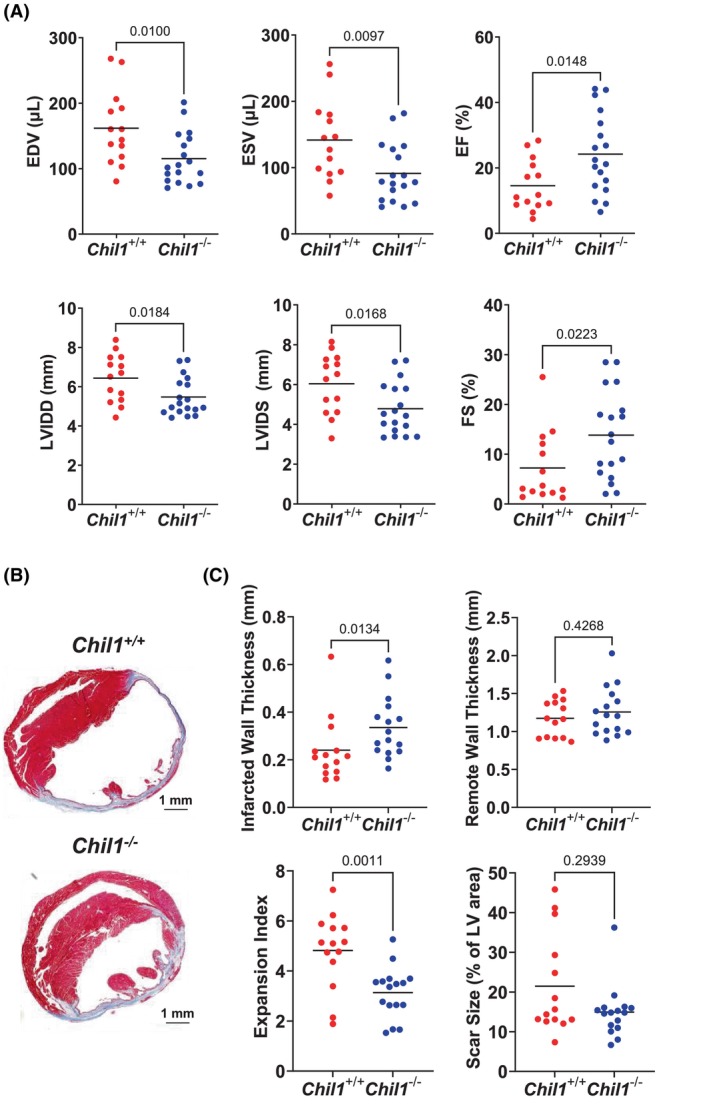
CHI3L1 exacerbates cardiac dysfunction and ventricular remodeling at 35 days post‐mi. Chil1^+/+^ and Chil1^−/−^ mice were subjected to MI. LV function was measured by echocardiography at 35 days post‐MI (A). Heart sections were stained with trichrome (B) and subjected to morphometric analysis (C). Student's *t*‐test.

To determine if CHI3L1 impairs wound healing post‐MI, we subjected *Chil1* ^*+/+*^ and *Chil1* ^*−/−*^ mice to MI and quantified cardiac function by echocardiography and LV remodeling by histology. We found that *Chil1* ^*−/−*^ mice exhibited preserved EF, cardiac output (CO), and stroke volume (SV), compared to *Chil1* ^*+/+*^ at 7 days post‐MI (Figure S4). Additionally, *Chil1* ^*−/−*^ mice had reduced ESV compared to *Chil1* ^*+/+*^ controls (Figure S4), but no differences in HR or EDV (Figure S4). These data support our hypothesis that CHI3L1 prolongs inflammation, exacerbates ventricular remodeling, and results in chamber dilation and dysfunction. Next, we assessed the extent to which CHI3L1 contributes to long‐term ventricular remodeling after MI. We found that *Chil1* ^*−/−*^ mice had increased EF at 35 days post‐MI relative to *Chil1* ^*+/+*^ controls and displayed decreased EDV, ESV, left ventricular internal dimensions in diastole (LVIDD), and left ventricular internal dimensions in systole (LVIDS) compared to *Chil1* ^*+/+*^ controls (Figure 5A), indicating increased chamber dilation in *Chil1* ^*+/+*^ mice. Furthermore, fractional shortening (FS) was increased in *Chil1* ^*−/−*^ mice (Figure 5A). These data indicate that ablation of CHI3L1 limits cardiac dilation and dysfunction after MI. To elucidate the effects of CHI3L1 signaling in collagen deposition and wound healing post‐MI, we subjected *Chil1* ^*+/+*^ and *Chil1* ^*−/−*^ mice to MI and used histology to quantify collagen deposition, cardiomyocyte hypertrophy, and capillary density. We first sought to elucidate the effects on scar formation by staining heart tissue sections with Masson's Trichrome. The data revealed that *Chil1* ^*−/−*^ mice exhibited increased infarcted wall thickness and reduced scar expansion index relative to *Chil1* ^*+/+*^ control mice (Figure 5B). Scar size as a percent of LV area and remote wall thickness remained unchanged between *Chil1* ^*+/+*^ and *Chil1* controls^*−/−*^ (Figure 5C). These data were further corroborated by echocardiographic assessment of anterior wall thickness and relative wall thickness parameters (Figure S5).

### 3.8 CHI3L1 exacerbates cardiac remodeling without effect on cardiomyocyte hypertrophy and capillary density

**FIGURE 6:**
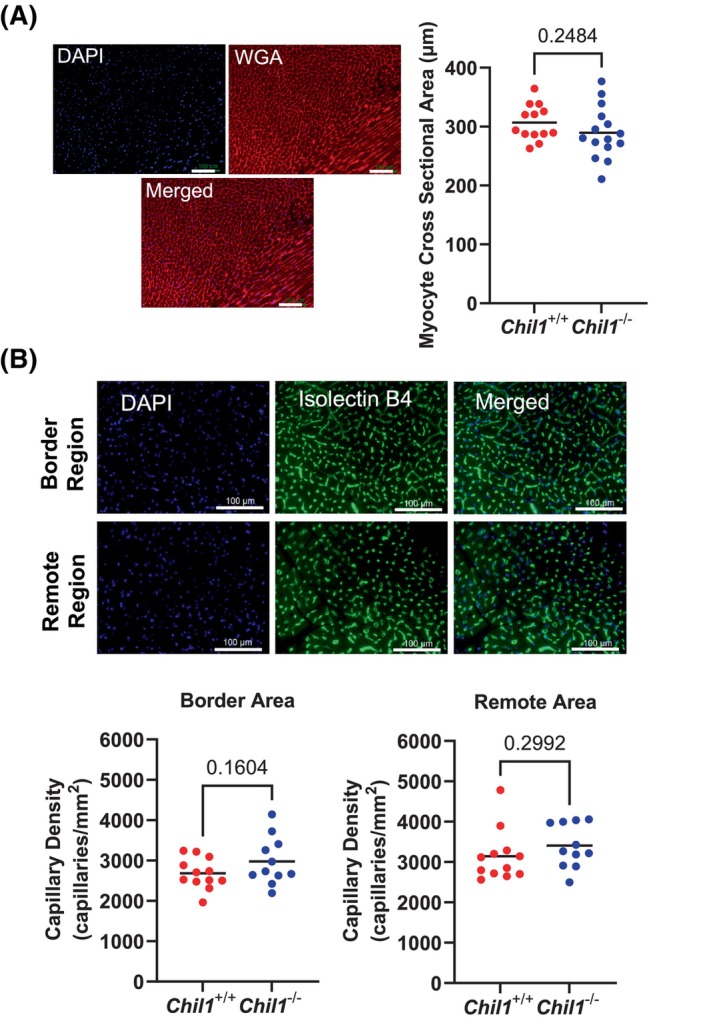
CHI3L1 exacerbates cardiac dysfunction post‐MI without effect on cardiomyocyte hypertrophy and capillary density. Chil1^−/−^ and Chil1^+/+^ mice were subjected to MI. After 5 weeks, heart sections were stained with DAPI and WGA, and images were assessed to evaluate myocyte cross‐sectional area and quantified (A). Heart tissues were stained with isolectin B4 and DAPI to visualize capillaries and nuclei, respectively. Capillary counts were quantified in border and remote areas of the LV (B). Student's *t*‐test.

To quantify cardiomyocyte hypertrophy post‐MI, 35 days post‐MI hearts from *Chil1* ^*+/+*^ and *Chil1* ^*−/−*^ mice were stained with WGA and DAPI. Cardiomyocyte cross‐sectional area analysis revealed no differences between *Chil1* ^*+/+*^ and *Chil1* ^*−/−*^ mice, indicating no difference in cardiomyocyte hypertrophy (Figure 6A). We further assessed LV remodeling at 35 days post‐MI by quantifying capillary density with Isolectin B4 staining. We found no differences in capillary density in either the border zone or remote zone of *Chil1* ^*−/−*^ or *Chil1* ^*+/+*^ mice (Figure 6B), indicating that CHI3L1 is not involved in angiogenesis post‐MI. Collectively, these data show that CHI3L1 contributes to scar expansion but not cardiomyocyte hypertrophy or capillary density post‐MI.

## 4 DISCUSSION

CHI3L1 is a biomarker associated with inflammation. Several clinical studies show that the plasma level of CHI3L1 increases after MI and remains elevated in chronic heart failure. Also, associative studies in patients show that the level of CHI3L1 in circulation inversely correlates with the recovery of the ventricular function after MI; however, the causative role of CHI3L1 in the pathophysiology of heart failure had been unknown.^12^, ^13^, ^14^, ^15^, ^16^, ^17^, ^18^, ^19^, ^20^, ^21^ In the current study, we showed that CHI3L1 increases in hearts after MI and is enriched in infarcted regions of the ventricle. We showed that neutrophils release CHI3L1, and it hinders pro‐reparative macrophage polarization in vitro. These data support in vivo observations that CHI3L1 perpetuates cardiac inflammation with an increase in pro‐inflammatory neutrophil and monocyte recruitment, which typifies non‐resolving inflammation. Finally, our study shows that CHI3L1 exacerbates ventricular remodeling after MI. Hence, these findings indicate that CHI3L1 is not only a reliable biomarker and predictor of heart failure but also contributes to ventricular remodeling and dysfunction.

CHI3L1, like other inflammatory markers (e.g., CRP, IL‐1, or TNF‐α) is not disease‐specific. In clinical populations, CHI3L1 is elevated in patients experiencing inflammation, tissue remodeling, and ongoing fibrosis.^15^, ^46^, ^47^ Numerous cells have been reported to secrete CHI3L1, including activated neutrophils, macrophages, vascular smooth muscle cells, chondrocytes, and cancer cells.^48^, ^49^ Our data, however, indicate that neutrophils, among cell types relevant to the heart, have the most abundant expression of CHI3L1. Based on our direct comparison with macrophages, B and T cells, fibroblasts, vascular cells, and cardiomyocytes, we show that neutrophils express four orders of magnitude more CHI3L1 than any other cells. This is consistent with proteomic studies showing that CHI3L1 is the second most abundant protein expressed in neutrophil secondary granules and rapidly released upon activation.^50^ Kinetic studies of CHI3L1 expression in infarcted hearts further support the conclusion that neutrophils may be the main source of CHI3L1 after infarction. We found that CHI3L1 expression is rapidly elevated at the peak of neutrophil infiltration at 2 days and gradually declines at 7 and 35 days but remains elevated in infarcted regions of the ventricles as compared to non‐infarcted regions. This corresponds with the kinetics of neutrophils after MI, where the neutrophil number declines after MI but remains elevated even in the chronic phase after MI. Although it is possible that chronically after MI, the progressive accumulation of fibroblasts and myofibroblasts may contribute to elevated CHI3L1 expression in hearts, it is unlikely that acutely after MI, cells other than neutrophils contribute substantially to CHI3L1 expression.

Others have suggested a salutary role for CHI3L1 post‐MI through promoting angiogenesis and fibroblast proliferation. According to Ye et al., overexpression of *Chil1* results in increased fibroblast activation and collagen deposition.^51^ Of note, their study overexpressed *Chil1* in cardiomyocytes. In our study, we showed that cardiomyocytes are not a significant source of CHI3L1 compared to neutrophils. The overexpression of *Chil1* in a cell type not normally expressing it may present as a confounding variable. We showed that the expression of CHI3L1 is significantly changed in only the ischemic region of the heart post‐MI. Hence, overexpression of *Chil1* in cardiomyocytes may force an unnatural pattern of CHI3L1 expression in the infarcted myocardium. Additionally, our gain‐of‐function approach involving the systemic administration of exogenous CHI3L1 in mice following myocardial infarction (MI) more closely resembles clinical scenarios, where elevated plasma levels of CHI3L1 are associated with poorer outcomes in patients with MI‐induced heart failure. More importantly, our strategy of deleting *Chil1* allows examination of the role of endogenously generated CHI3L1 in infarcted hearts. Another study promoted a role for CHI3L1 in promoting angiogenesis in myocardial infarction; however, those studies investigated only CHI3L1's potential to drive angiogenesis in vitro and not natively in hearts post‐MI.^52^ In our study, we found no role for CHI3L1 in angiogenesis in the remote region of the heart post‐MI. Because we show that CHI3L1 expression does not change in the remote regions post‐MI, our studies reveal that CHI3L1 does not natively display salutary effects in hearts after MI.

Macrophages play a central role in orchestrating wound healing after MI. There are two developmentally distinguished macrophage populations in the heart.^2^, ^9^ The cardiac resident CCR2^Neg^ macrophages deposited during development and monocyte‐derived CCR2^Pos^ macrophages are recruited to the heart after ischemic injury.^2^, ^10^, ^53^, ^54^ Furthermore, LyC6^high^ monocyte‐derived macrophages, upon activation, can acquire a pro‐inflammatory or pro‐reparative/resolving phenotype. Pro‐inflammatory macrophages produce proinflammatory cytokines and perpetuate inflammation, while pro‐reparative macrophages produce pro‐resolving and anti‐inflammatory cytokines and bioactive lipids, as well as cytokines facilitating recruitment, proliferation, and activation of matrix‐producing fibroblasts.^2^, ^55^ Thus, pro‐inflammatory macrophages will antagonize, while M2 will promote, wound healing.^2^ Neutrophils can guide both pro‐inflammatory and pro‐reparative macrophage phenotypes. Neutrophils produce pro‐inflammatory cytokines, anti‐microbial proteins, and DAMPs, which primarily drive the pro‐inflammatory phenotype.^56^ Nevertheless, some of the granule content and efferocytosis of dead neutrophils promote pro‐reparative polarization.^2^, ^7^ We found that *Chil1* ^−/−^ mice showed decreased neutrophils and Ly6C^High^ monocytes in hearts at 5 days after MI compared to their wild‐type littermate controls, suggesting faster resolution of inflammation in the absence of CHI3L1. Thus, we posit that CHI3L1 antagonizes wound healing and promotes ventricular remodeling via inhibition of the pro‐reparative phenotype in macrophages. This hypothesis was supported by our data showing that *Chil1* ^*−/−*^ mice exhibit preserved ventricular volumes and function at 7 days and sustained preservation of remodeling and function at 35 days after MI compared to their *Chil1* ^+/+^ controls. Finally, the gain‐of‐function experiment in wild‐type mice showed that exogenous CHI3L1 administration promotes ventricular remodeling and dysfunction at 35 days after MI. These data corroborate data in patients indicating that the plasma level of CHI3L1 in patients at the time of MI inversely correlates with recovery of cardiac function during the follow‐up period. Furthermore, a heightened level of CHI3L1 is a strong predictor of reinfarction and mortality in patients with HF due to MI.^12^, ^13^, ^14^, ^15^, ^16^, ^17^, ^18^, ^19^, ^20^, ^21^ Nevertheless, our in vitro experiments show that recombinant CHI3L1 has no direct effect on macrophage polarization based on gene expression profile. These data suggest that the immunomodulatory effects of CHI3L1 are independent of direct signaling in macrophages.

In summary, we showed that elevated CHI3L1 in infarcted regions of the hearts after MI perpetuates non‐resolving inflammation and contributes to ventricular remodeling. Although we do not know the exact molecular mechanism whereby CHI3L1 governs prolonged inflammation after MI, it appears that inhibition of CHI3L1 may be beneficial to limit cardiac dysfunction in acute and chronic heart failure due to MI. This could be explored as a potential druggable target in patients.

## AUTHOR CONTRIBUTIONS

*Methodology, validation, formal analysis, investigation, writing—original draft*: Jonah K. Stephan. *Methodology, validation, formal analysis, investigation*: Taylor Knerr, Zhen Gu, Hong Li, Kenneth R. Brittian, Sujith Dassanayaka and Richa Singhal. *Methodology, investigation*: Yibing Nong. *Design, interpretation, funding, writing, revising*: Steven P. Jones. *Conceptualization, design, interpretation, funding, writing, revising, methodology, validation, formal analysis*: Marcin Wysoczynski.

## FUNDING INFORMATION

This work was supported by the National Institutes of Health (grant numbers R01 HL141191, P30 GM127607, S10 OD025178, F31 HL174069) and the American Heart Association (AHA PRE1243117).

## DISCLOSURES

The authors have nothing to disclose.

## Supporting information


**Supplemental Figure 1.** Neutrophils are the main source of CHI3L1. Whole bone marrow cell fraction was subjected to scRNAseq and unbiased clustering to identify individual cell populations. Chil1 expression coincides with neutrophil Ly6g marker (A). Neutrophils, macrophages, fibroblasts, endothelial cells, cardiomyocytes, B cells, and T cells were assessed for CHI3L1 expression with ELISA. *N* = 3, one‐way ANOVA (B). Neutrophils were sorted from the bone marrow and culture in control media or the presence of A23178 Ionophore, LPS, and PMA. At 10–180 min conditioned media was quantified for CHI3L1 levels with ELISA. *N* = 3, two‐way ANOVA (C).

**Supplemental Figure 2.** CHI3L1 has no effect on medullary and extramedullary granulopoiesis after MI. Mice were acutely subjected to MI and 2 days later bone marrow and spleens were probed with fluorescently labeled antibodies. Neutrophils were quantified as CD11b^Pos^Ly6G^Pos^. Immature neutrophils were identified as CD101^Neg^ and mature as CD101^Pos^. Total neutrophils in the bone marrow were quantified per femur (A) and per mg in the spleen (B) and stratified to mature and immature neutrophils in the bone marrow (C) and spleen (D). Student’s *t*‐test.

**Supplemental Figure 3.** Recombinant CHI3L1 has no effect on macrophage polarization in vitro. Stimulation with LPS (100 ng/mL) and IFN‐γ (20 ng/mL) induced M1 polarization in bone marrow derived macrophages (A) and peritoneal macrophages (B) in the presence of recombinant CHI3L1. Stimulation with IL‐4 (10 ng/mL) and IL‐13 (10 ng/mL) to induce M2 Polarization in bone marrow derived macrophages (C) and peritoneal macrophages (D) in the presence of recombinant CHI3L1. Two‐Way ANOVA with Multiple Comparisons.

**Supplemental Figure 4.** CHI3L1 deficiency preserve cardiovascular function at 7 days post‐MI. *Chil1* ^*+/+*^ and *Chil1* ^*−/−*^ mice were subjected to MI and cardiovascular function was measured by echocardiography at 7 days post‐MI. Student’s *t*‐test.

**Supplemental Figure 5.** CHI3L1 deficiency preserves LV structure measures with echocardiography. *Chil1* ^*+/+*^ and *Chil1* ^*−/−*^ mice were subjected to MI and cardiovascular function was measured by echocardiography at 35 days post‐MI. Student’s *t*‐test.

## Data Availability

The data that support the findings of this study are available in the Materials and Methods, Results, and/or Supplemental Material of this article.
