# Supplementary figures and images for: Neutrophil‐secreted CHI3L1 exacerbates cardiac dysfunction and inflammation after myocardial infarction
Source: FASEB J. 2025 Feb 27;39(5):e70422. doi: 10.1096/fj.202401654R (PMC11963974; doi:10.1096/fj.202401654R)

# Supplemental Figure 1.

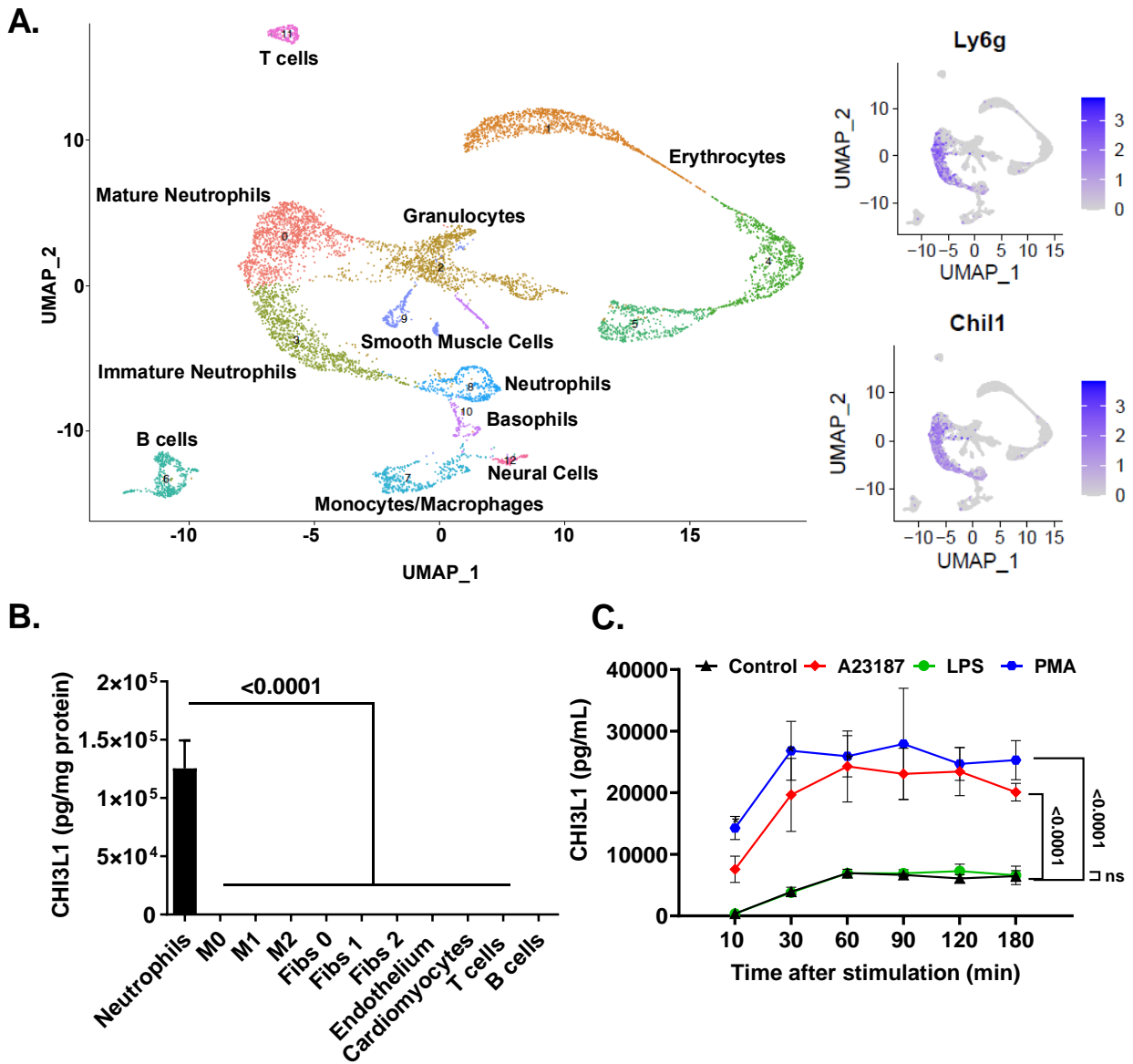

Supplement: Supplementary file 1 — Supplemental Figure 1. Neutrophils are the main source of CHI3L1. Whole bone marrow cell fraction was subjected to scRNAseq and unbiased clustering to identify individual cell populations. Chil1 expression coincides with neutrophil Ly6g marker (A). Neutrophils, macrophages, fibroblasts, endothelial cells, cardiomyocytes, B cells, and T cells were assessed for CHI3L1 expression with ELISA. N = 3, one‐way ANOVA (B). Neutrophils were sorted from the bone marrow and culture in control media or the presence of A23178 Ionophore, LPS, and PMA. At 10–180 min conditioned media was quantified for CHI3L1 levels with ELISA. N = 3, two‐way ANOVA (C). [file FSB2-39-e70422-s003.pdf]

# Supplemental Figure 2.

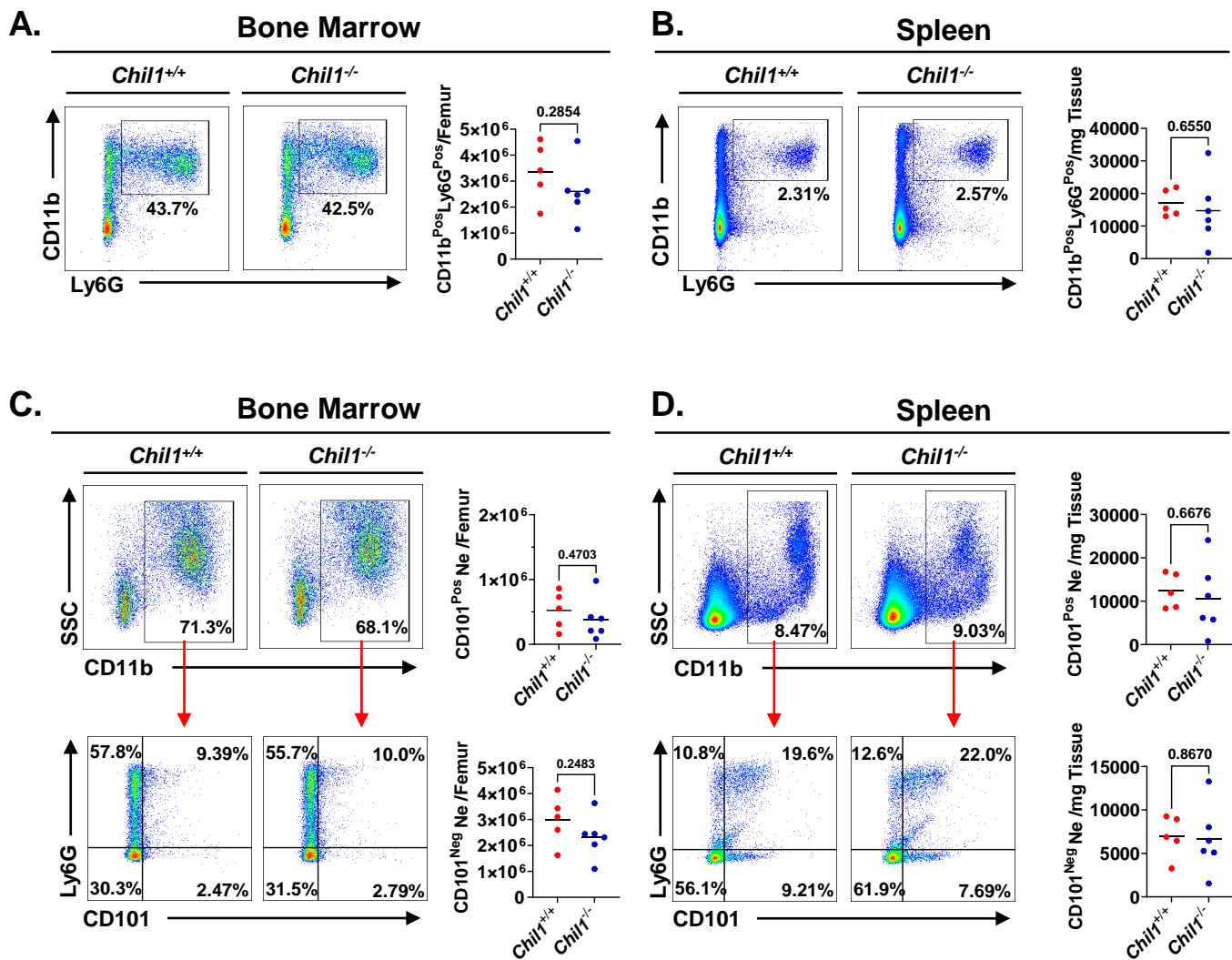

Supplement: Supplementary file 2 — Supplemental Figure 2. CHI3L1 has no effect on medullary and extramedullary granulopoiesis after MI. Mice were acutely subjected to MI and 2 days later bone marrow and spleens were probed with fluorescently labeled antibodies. Neutrophils were quantified as CD11bPosLy6GPos. Immature neutrophils were identified as CD101Neg and mature as CD101Pos. Total neutrophils in the bone marrow were quantified per femur (A) and per mg in the spleen (B) and stratified to mature and immature neutrophils in the bone marrow (C) and spleen (D). Student’s t‐test. [file FSB2-39-e70422-s002.pdf]

# Supplemental Figure 4.

7 d

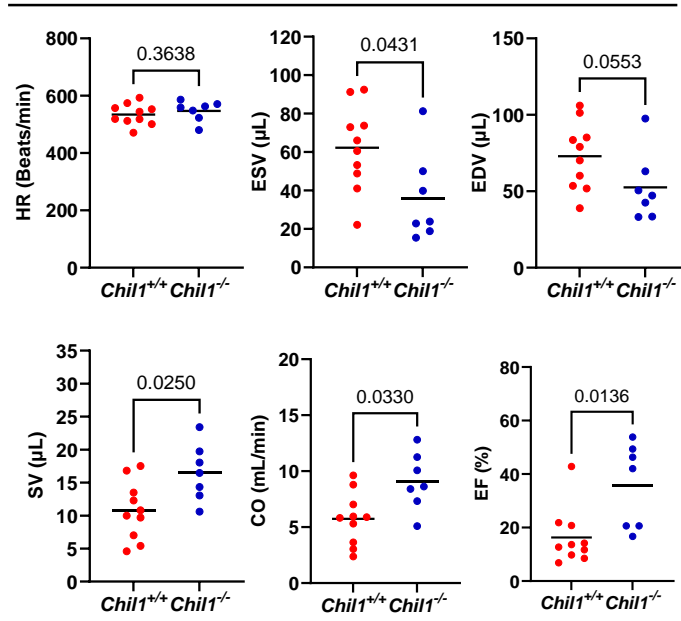

Supplement: Supplementary file 4 — Supplemental Figure 4. CHI3L1 deficiency preserve cardiovascular function at 7 days post‐MI. Chil1 +/+ and Chil1 −/− mice were subjected to MI and cardiovascular function was measured by echocardiography at 7 days post‐MI. Student’s t‐test. [file FSB2-39-e70422-s004.pdf]

# Supplemental Figure 5.

35 d

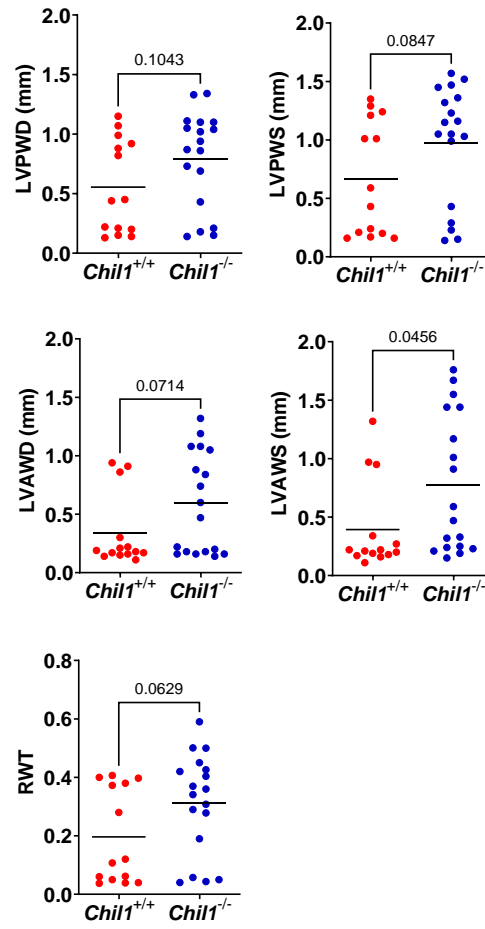

Supplement: Supplementary file 5 — Supplemental Figure 5. CHI3L1 deficiency preserves LV structure measures with echocardiography. Chil1 +/+ and Chil1 −/− mice were subjected to MI and cardiovascular function was measured by echocardiography at 35 days post‐MI. Student’s t‐test. [file FSB2-39-e70422-s001.pdf]
