# Supplementary material for: Neutrophil‐secreted CHI3L1 exacerbates cardiac dysfunction and inflammation after myocardial infarction
Source: FASEB J. 2025 Feb 27;39(5):e70422. doi: 10.1096/fj.202401654R (PMC11963974; doi:10.1096/fj.202401654R)

Supplemental Figure 3.

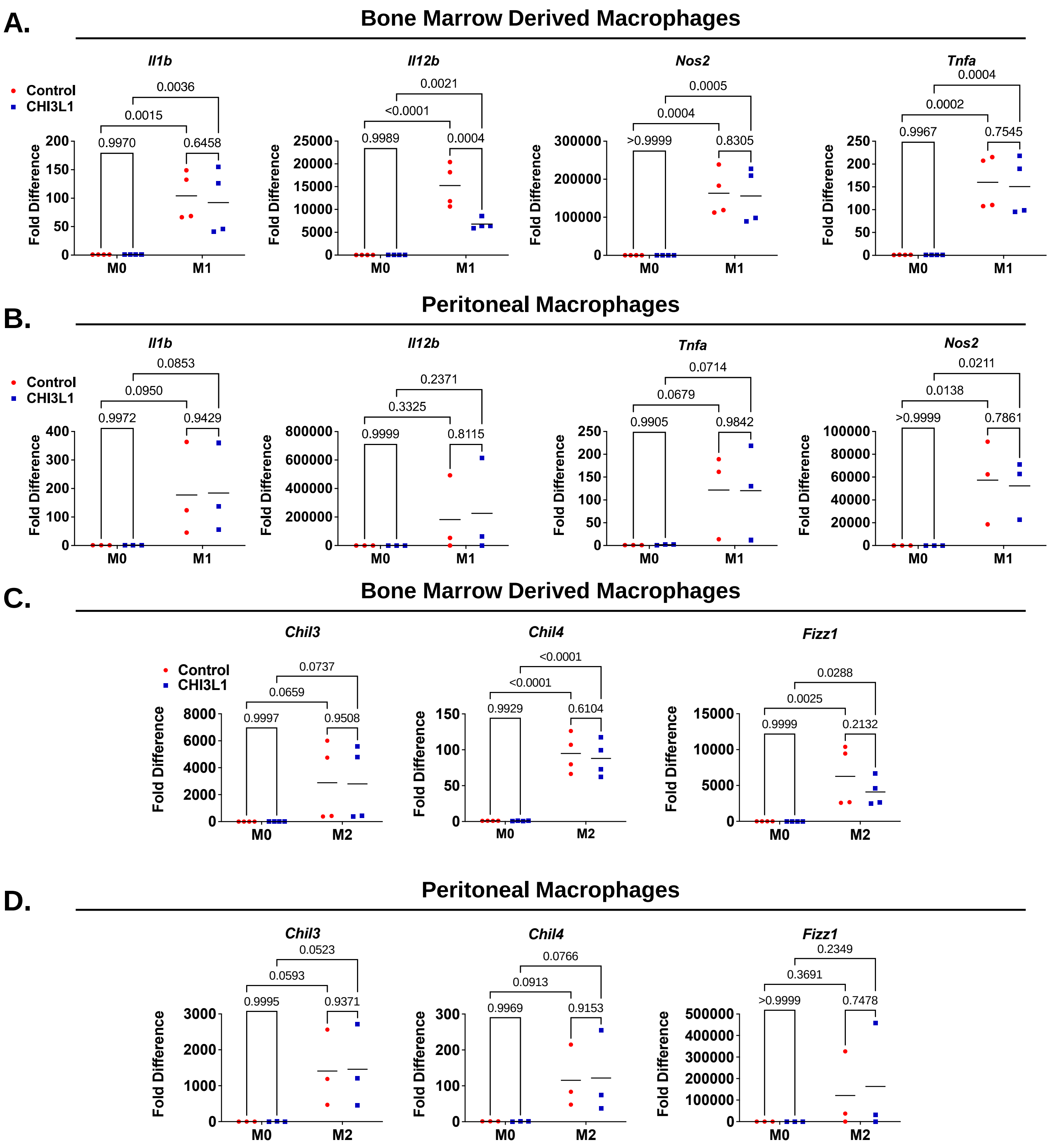

Supplement: Supplementary file 3 — Supplemental Figure 3. Recombinant CHI3L1 has no effect on macrophage polarization in vitro. Stimulation with LPS (100 ng/mL) and IFN‐γ (20 ng/mL) induced M1 polarization in bone marrow derived macrophages (A) and peritoneal macrophages (B) in the presence of recombinant CHI3L1. Stimulation with IL‐4 (10 ng/mL) and IL‐13 (10 ng/mL) to induce M2 Polarization in bone marrow derived macrophages (C) and peritoneal macrophages (D) in the presence of recombinant CHI3L1. Two‐Way ANOVA with Multiple Comparisons. [file FSB2-39-e70422-s005.pdf]
